# Young Women’s Perspectives on Alcohol Environments in Urban Informal Settlements in Kampala, Uganda: An Exploratory Photovoice Analysis

**DOI:** 10.3390/ijerph23080975

**Published:** 2026-07-28

**Authors:** Allisa George, Jacqueline Nassaka, Anna Nabulya, Jane Palmier, Emeka W. Dumbili, Monica H. Swahn

**Affiliations:** 1 Department of Health Promotion and Physical Education, Wellstar College of Health and Human Services, Kennesaw State University, Kennesaw, GA 23219, USA; 2 Uganda Youth Development Link, Kampala P.O. Box 12659, Uganda; 3 School of Public Health, Virginia Commonwealth University, Richmond, VA 23284, USA; 4 School of Sociology, College of Social Sciences and Law, University College Dublin, Belfield, D04 V1W8 Dublin, Ireland

**Keywords:** alcohol environments, social determinants of health, photovoice, urban informal settlements, gender, mental health

## Abstract

**Highlights:**

**Public health relevance—How does this work relate to a public health issue?**

- Young women described alcohol environments in urban informal settlements as contributing to violence, psychological distress, and social inequities.
- This study highlights the potential importance of alcohol as a social and commercial determinant of health in low-resource settings where regulatory protections are limited.

**Public health significance—Why is this work of significance to public health?**

- This work provides exploratory participatory evidence on how young women perceive and experience alcohol environments in urban informal settlements.
- This work highlights the perceived dual role of alcohol environments as supporting livelihoods and social interaction while also being associated with risk and harm.

**Public health implications—What are the key implications or messages for practitioners, policy makers and/or researchers in public health?**

- These exploratory findings identify alcohol availability, marketing, regulation, and gender-responsive approaches as areas warranting further investigation.
- Community-driven participatory approaches may help inform future alcohol research and intervention development that considers both economic realities and health risks.

**Abstract:**

Young women living in urban informal settlements experience intersecting structural and environmental conditions that shape health and well-being. Alcohol environments (the physical, social, and commercial contexts in which alcohol is sold, marketed and consumed) represent an important but understudied social and commercial determinant of health. Young women in these settings face intersecting vulnerabilities, including poverty, unemployment, and gender inequality, that increase their exposure to alcohol-related harm. Although alcohol use and alcohol-related harms are increasingly understood as shaped by social and commercial determinants of health, little is known about how young women experience alcohol environments in low-resource settings. This exploratory, site-specific secondary, qualitative analysis draws on data from a 2022 Photovoice project conducted over several weeks with 15 young women (ages 18–24) living in urban poverty in Kampala. Participants used photography and group discussions to explore how alcohol shapes their lives. Our inductive thematic analysis included all five participants from the Makindye site, where alcohol-related photographs and narratives emerged prominently during the Photovoice discussions. Six key themes were identified from 42 narrative excerpts and two photographs, highlighting alcohol’s dual role as both a source of livelihood and a source of harm. Participants described pervasive exposure to aggressive marketing, unregulated drinking venues, and the normalization of alcohol use, often linked to interpersonal violence, emotional distress, and disruption of household dynamics. Alcohol environments also constrained women’s mobility and safety, limiting their participation in public life. These findings suggest that alcohol environments may shape young women’s exposure to violence, economic precarity, and psychological distress in this setting. They also point to the potential value of gender-responsive policies, stronger regulation, and community-based prevention strategies to address alcohol-related harm and health inequities in low-resource urban settings.

## 1. Background

Alcohol use and alcohol-related harm are increasingly recognized as pressing global concerns, particularly in low- and middle-income countries (LMICs) where health systems often lack the capacity to respond effectively [1,2]. More commonly, alcohol use is increasingly understood through a social determinant of health framework that highlights how structural, environmental, and commercial forces shape alcohol consumption and other health risks and harms.

In Kampala’s urban informal settlements, social determinants of alcohol use and harm include overcrowded living conditions, homelessness, poor sanitation, limited access to safe spaces, exposure to violence, and pervasive presence of alcohol [3,4,5]. Together, these conditions are associated with anxiety, depression, psychosocial distress and suicidality [6,7,8] and alcohol-related harm, yet remain under-addressed in research, treatment and policy. Alcohol and other drug use may also serve as maladaptive coping mechanisms, providing temporary relief while potentially increasing vulnerability to further harms [4,9,10,11,12].

Alcohol use and alcohol-related harms are shaped by both social and commercial determinants of health [13,14]. Its widespread availability is driven by dense outlet concentration, limited regulation, and aggressive marketing practices that normalize consumption, especially among young people and women in emerging markets [15,16,17]. While often portrayed as a symbol of empowerment or social status as part of marketing efforts, alcohol use is linked to increased risks of interpersonal violence, financial strain, and adverse mental health outcomes [12,18,19,20].

Uganda presents a critical context for examining these dynamics. The country ranks among the highest in sub-Saharan Africa with 12.2 L per capita alcohol consumption per year [2], yet alcohol policy and regulation remain weak or inconsistently enforced [21,22,23]. Although alcohol taxation contributes substantially to national revenue, tax rates remain relatively low compared to WHO recommendations and thus offer a limited deterrent effect and public health protection [23,24,25]. This creates broader tension where the alcohol industry is economically important, but its growth exacerbates social and health consequences, including alcohol-related harm, productivity loss and added pressure on health and social welfare systems [21]. In a setting with limited regulatory capacity, these competing interests make it difficult to effectively mitigate alcohol-related harm [26].

Previous research has shown that youth living in Kampala’s informal settlements are routinely exposed to alcohol marketing through branded advertisements, community events, and informal vendors [27,28], but research measuring alcohol marketing exposure is scarce [29]. Even so, research in this setting demonstrates that exposure to alcohol marketing encourages early alcohol use initiation and reinforces social norms that blur the lines between recreation and risk, especially for women and girls [30,31]. It is within this context that alcohol use and related harms are prevalent among young people in Kampala [3,4,20] and encompass serious violence, sexual victimization child maltreatment, suicidal ideation, and HIV risk [4,7,32,33,34,35].

Despite growing attention to alcohol’s role in health inequities, few studies have explored how young women in informal urban settings perceive and experience the alcohol environment [28], and there is a clear need to advance alcohol research in these settings [36]. In this study, we use the term “alcohol environment” to refer to the physical, social, and commercial features of everyday settings that shape alcohol availability, visibility, promotion, consumption, and related harms. These features may include alcohol outlets and informal sales, advertising and branding, drinking-related activities, social norms, and the ways alcohol-related places intersect with community safety, livelihoods, and well-being. In this context, this exploratory analysis examines how young women in the Makindye community in Kampala perceived and experienced alcohol environments and the ways these environments intersected with their safety, livelihoods, and well-being. Moreover, participatory approaches that elevate the voices and lived realities remain limited, although some studies have used participatory methods to determine alcohol availability and marketing, thus generating policy recommendations, and to reduce alcohol use disorders and harm [36,37,38,39].

In response to the alcohol-related concerns that emerged during participatory discussions of this Photovoice project, the current analysis examines how young women at one study site in Kampala experienced and interpreted the alcohol environment, and the harms they associated with it in relation to their mental health and well-being. Drawing on Photovoice, a participatory visual research method that combines photography with collective dialogue, we explore these questions as part of the TOPOWA Project (TOPOWA means to never give up in Luganda, a local language in Uganda). This analysis was part of a larger Photovoice project where young women living in three informal settlements in Kampala documented the linkages between space and mental health. The overall multicomponent TOPOWA project was designed to examine multidimensional threats to young women’s mental health and well-being using the Research Domain Criteria Framework [40].

Previous research in Kampala has documented alcohol use, alcohol-related harm, and exposure to alcohol marketing. However, less is known about how young women living in informal settlements experience and interpret alcohol-related places in their everyday lives, including their overlapping social, economic, and commercial functions and their perceived implications for safety and well-being. This exploratory Photovoice analysis addresses that gap by examining participant-generated photographs and discussions from the Makindye study site. The key purpose of the larger Photovoice Project was to determine how the young women defined and conceptualized mental health and well-being. Also, to determine if there is a link between place, mental health and well-being. Alcohol was not a predefined analytic focus of the broader Photovoice project; rather, it emerged organically during group discussions at the Makindye site. Although the broader Photovoice project included three study sites, only participants in Makindye raised alcohol as a dominant theme in their photographs and discussions. Focusing on this subset allows us to explore in depth how alcohol environments shape young women’s safety, well-being, and mental health in a low-resource urban setting.

## 2. Methods

The Photovoice project was implemented across three study sites in metropolitan Kampala, Uganda, with 15 young women aged 18 to 24 years participating between August and September 2022. Photovoice was selected because it enables participants to document and interpret features of their environment through photographs and discussion, making it well suited for exploring lived experiences that may be difficult to capture through conventional interviews alone. Participants were recruited from Youth Support Centers operated by the Uganda Youth Development Link (UYDEL). UYDEL is a community-based organization seeking to uplift youth through psychosocial services and intervention programs. Participants were informed that researchers from several universities were conducting a study to understand young women’s experiences, their communities, and how environmental factors influence well-being and mental health. The broader TOPOWA Photovoice Project has generated several publications examining young women’s mental health and well-being across study sites. The present paper represents a distinct, site-specific analysis focused on alcohol environments, which have not been examined in prior publications from this project. Detailed descriptions of the methodology and key project findings have been published elsewhere [40,41,42,43,44].

### 2.1. Sampling and Recruitment

Participants were recruited by word of mouth through the UYDEL drop-in centers and self-selected into the study after providing written informed consent. Ethical approvals were obtained from all relevant institutional review boards. Although the broader Photovoice project enrolled 15 young women across three UYDEL sites, this analysis focuses solely on the five participants at the Makindye site, where alcohol-related themes were identified during the Photovoice activities.

### 2.2. Methods of Data Collection and Project Implementation

The Photovoice activities were facilitated by two African women affiliated with UYDEL who were culturally and linguistically aligned with participants and fluent in both English and Luganda. Both facilitators were certified through CITI training and were experienced in community-based work, with one having specific training in participatory photography. The Principal Investigator also received training in the Photovoice approach. Several co-authors of this article were directly involved in the study implementation and data collection, while additional authors joined post–data collection for analytic interpretation under the guidance and approval of the Project Principal Investigator.

The Photovoice project consisted of five sessions. All participants took part in a one-day group training and practice session that introduced the purpose of the study, taught participants how to take photographs using a smartphone, and covered ethical and safety considerations for photographing in the community. The session also provided an opportunity for participants to connect, practice, and ask questions. Additional methodological details have been described elsewhere [40,41,42,43,44]. Participants were then asked to capture photographs that reflected the concept of “TOPOWA,” meaning “do not give up,” and to depict what mental health and well-being meant to them, as well as how place, mental health, and well-being might be interconnected. The initial training was followed by three weekly small-group sessions at each of the three study sites with the five women from that specific site. During these sessions, participants reviewed and discussed their photographs in response to guiding prompts. The final session was a one-day large-group theme validation meeting with all participants from the three study sites. During the weekly sessions, participants were trained and asked to prepare responses to specific discussion questions (Table 1).

The discussion questions were adapted from the SHOWeD framework commonly used in Photovoice research [45,46]. During the final session, participants and facilitators reviewed the photographs in a gallery-style workshop, discussed and refined the emerging thematic areas, and collaboratively grouped the photographs taken across the three study sites and labeled themes and subthemes. This participatory process supported collective interpretation and validation of the findings.

Each participant was provided with a Samsung A03 smartphone (Seoul, Republic of Korea) to take photographs during the project period, with the device’s GIS detection enabled for weekly discussion meetings. Smartphones were selected because they were less conspicuous in the community and allowed for geo-coded photos without the additional cost of a separate GPS device. All phones were collected by the study team at the conclusion of the project. Participants received training on applicable ethical and safety considerations when taking photos in the community and were discouraged from photographing identifiable individuals. Instructions were provided for obtaining consent when necessary. The training also covered how to caption photographs and convey their intended messages. The day concluded with a guided community visit and field practice session focused on taking photographs, framing images, and navigating the community setting. Finally, participants received light refreshments during each meeting. As reimbursement for transportation and time, participants were provided $24 following the one-day training session, $8 after the follow-up training, and $8 after each fortnightly meeting. Participants who completed all components of the project (five meetings in total) received a total of $56 in compensation.

### 2.3. Methods of Data Analysis

This analysis included the data provided by all five participants enrolled at the Makindye site, where alcohol-related photographs and narratives emerged prominently across the Photovoice discussions. We reviewed the Photovoice materials generated at the Makindye site, including participant photographs, captions, and associated group discussions, and identified narrative excerpts related to alcohol environments, including bars and entertainment venues. Because only two alcohol-related photographs were available, the analysis relied primarily on the associated captions and group-discussion narratives, with the photographs serving as contextual visual material. Accordingly, the present study is best understood as a qualitative thematic analysis of narratives that emerged from the broader Photovoice project, with the photographs serving as contextual anchors rather than as a separate visual dataset. The 42 alcohol-related narrative excerpts were examined in relation to their photographic and discussion context and organized into six thematic categories reflecting participants’ experiences of alcohol-related spaces in their lives and communities. The analysis followed an inductive, iterative approach consistent with qualitative thematic methods. Two members of the research team independently reviewed the 42 quotations from Makindye and manually grouped them into preliminary categories based on recurring ideas and patterns. In addition to identifying recurring topics, the researchers examined relationships and tensions across the categories, including the ways alcohol-related spaces were described simultaneously as sources of livelihood, social activity, insecurity, and harm. The researchers then compared their groupings, discussed discrepancies, and refined the categories through consensus. This manual process supported consistency in interpretation and helped ensure that the themes remained grounded in participants’ accounts. Final categories were reviewed by the broader research team to enhance analytic rigor. Several members of the research team were involved in the broader Photovoice project, while others joined specifically for the present analysis and brought additional expertise in alcohol research and qualitative interpretation. To reduce the influence of individual assumptions, two researchers independently reviewed the materials, compared interpretations, resolved differences through discussion, and sought to keep the themes grounded in participants’ photographs and narratives.

## 3. Results

Study participants were between 18 and 24 years of age. None of them were married, and their educational attainment ranged from Secondary 2 to Secondary 4. Four participants identified as Christian and one as Muslim. None were mothers. Two participants were employed, reporting monthly earnings between 120,000 and 200,000 Uganda Shillings (approximately $32 to $54), reflecting their limited socioeconomic resources. The participants reported employable skills such as hairdressing, tailoring, and electrical installation. Their household sizes ranged from one to seven members. All five participants contributed alcohol-related reflections, resulting in 42 alcohol-focused quotations. To protect confidentiality, all participants used pseudonyms.

**Figure 1:**
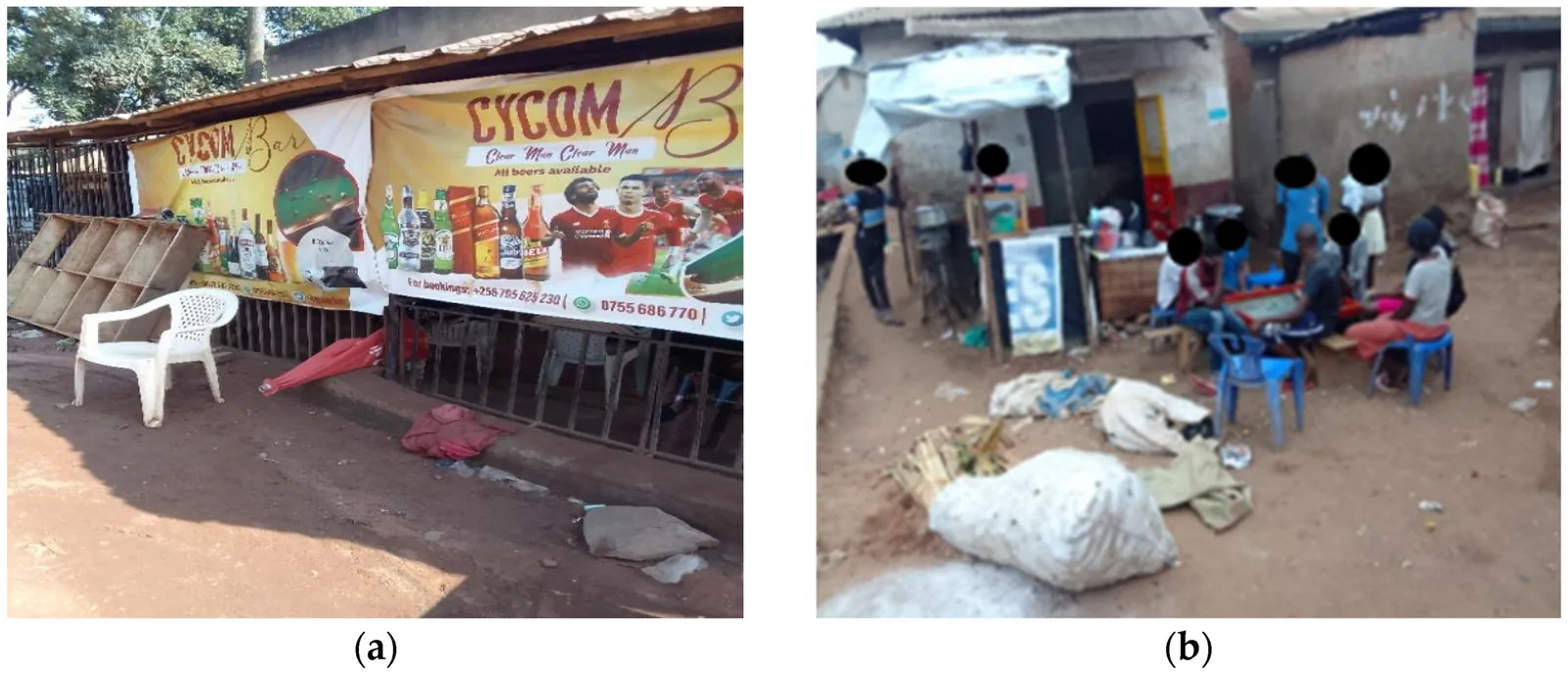
Photo (**a**) by Patience, 23 years, and Photo (**b**) by Mpambe, 18 years. Depicting alcohol environment. Photo (**a**): “Leisure places help reduce stress in slum communities”. Photo (**b**): “I want to show that there are drunkards in our community”.

The analysis focused on identifying how participants described and interpreted alcohol environments in relation to safety, daily routines, economic activity, and mental well-being, and on examining patterns and tensions that emerged across participants’ narratives. The analysis generated six interconnected themes based primarily on participants’ captions and group discussion narratives. The two alcohol-related photographs provided visual context for participants’ descriptions of alcohol-related spaces but were not analyzed as a separate visual dataset. Across the themes, participants’ accounts revealed several tensions, particularly the ways alcohol-related spaces were described as supporting recreation, social interaction, and livelihoods while also contributing to insecurity, financial strain, and harm. Although the themes are interrelated, each captures a distinct dimension of how alcohol environments were described to shape participants’ safety, mental health, economic activity, and daily life (see Table 2). Together, these themes offer a comprehensive framework for understanding participants’ experiences and the broader social determinants shaping alcohol-related harm (Figure 1 and Table 2).

### 3.1. Alcohol Use and Community Dynamics

Participants described alcohol as being deeply embedded in everyday community life, shaping leisure, social interaction, and perceptions of normalcy. Drinking was frequently linked to watching football, playing pool, and other recreational activities, reinforcing its routine presence in public spaces, as Figure 1a shows with the caption “*Leisure places help reduce stress in informal settlements communities*” (Patience, 23 years). The photograph prompted a broader group discussion about the role of alcohol-related spaces in the community. Across this discussion, participants described bars as both social and economic resources and sources of concern. Participants described these as places for watching football, playing pool, socializing, and relieving stress, while also associating them with heavy drinking, betting, fights, noise, and financial strain. As one participant explained, “*Many times we have different ways to deal with stress, some of us love sports and pool table games so we can visit the bar to watch football and socialize with friends, and this helps to strengthen social gatherings and togetherness in the community* (Lisa, 18 years). They noted that bars generated income for owners, attendants, food vendors, and nearby businesses, but also expressed concern about children’s exposure to drinking and other risky activities. Participants noted that individuals sometimes prioritized alcohol spending over household needs, contributing to economic instability. These accounts suggest that the benefits and harms associated with alcohol-related spaces were not viewed as separate. Rather, the same accessibility, social activity, and economic opportunities that made bars valued community spaces also increased exposure to drinking, financial loss, conflict, and other risks. Overall, participants positioned alcohol-related spaces as socially and economically embedded within community life and associated with both livelihood and harm.

### 3.2. Unemployment, Idleness and Risk

The discussion of Figure 1b extended this interpretation beyond alcohol use alone. Participants viewed the daytime gathering around the game space as visible evidence of limited employment opportunities and a lack of constructive activities. As Patience explained, “*Lots of idle people and this means that they may have limited job opportunities in their area [and] a lot of leisure time because of their unemployment*” (Patience, 23 years). In this account, idleness was understood not simply as an individual behavior, but as a reflection of constrained economic opportunities within the community. Participants frequently linked alcohol use to unemployment and limited opportunity. Idle time was described as drawing people into bars and game spaces during the day, reinforcing patterns of alcohol and drug use. One participant observed that “*most idle people spend time in bars*” (Hopebella, 19 years), interpreting this as a reflection of job scarcity. Idleness was also associated with theft, property damage, and nighttime crime. Participants also described a perceived progression from daytime idleness and substance use to nighttime insecurity. One participant noted that “*in the evening after taking alcohol they commit crimes and theft especially at night*” (Mbampe, 18 years). Figure 1b therefore prompted discussion of how unemployment, unstructured time, alcohol and drug use, and community insecurity were understood as interconnected. Alcohol-centered settings were therefore understood as both symptoms and reinforcers of structural precarity.

### 3.3. Alcohol Marketing and Accessibility

Participants expressed concern about the visibility and accessibility of alcohol, particularly for children and young people (Figure 1a). Bars were described as poorly enclosed and continuously operating, increasing exposure to drinking behaviors. As one participant noted, “*The bar is not closed well…children in this community may come and…see some of the other risky activities that take place in bars and some can learn these behaviours and start trying out or experimenting on drinking*” (Lisa, 18 years). Similarly, participants expressed concern about the visibility and accessibility of alcohol, particularly for children and young people, as illustrated by the poorly enclosed bar. The omnipresence of alcohol in community spaces reinforced its normalization despite awareness of health risks. Participants’ accounts suggested that accessibility operated not only through the physical availability of alcohol, but also through the routine visibility of drinking within everyday community life. Participants also recognized the economic dimension of alcohol commerce, noting that bars generate income for owners and workers. However, accessibility and constant operation were viewed as heightening vulnerability, especially for youth. This created a tension between the economic value of alcohol-related businesses and participants’ concerns about early exposure to alcohol marketing and use, normalization, and the potential adoption of risky behaviors among young people.

### 3.4. Alcohol, Violence, and Crime

Alcohol-centered spaces were frequently associated with insecurity, including fights, theft, and harassment. Participants described drinking environments as sites where conflicts escalate and property is damaged. For young women in particular, these settings were perceived as unsafe. One participant explained that as a young girl, she might fear harassment or theft in such spaces, noting, “*As a young girl, they will harass me, and I will go back with no money*” (Esther, 21 years). Gendered risks were especially salient, including pressure to seek employment in bars and potential exposure to transactional sex and substance use. As another participant noted, “*Idle people…after taking alcohol they commit crimes and theft especially at night*” (Mbampe, 18 years). These risks were intensified by limited employment opportunities, which could draw young women into alcohol-related settings for income while simultaneously increasing their exposure to exploitation and harm. Overall, alcohol-related spaces were understood as gendered environments that constrained women’s mobility and safety while reflecting their broader economic vulnerability.

### 3.5. Alcohol, Mental Health, and Well-Being

Alcohol use was described as both a temporary coping mechanism and a source of psychological strain. Some participants noted that visiting bars, listening to music, or watching football could provide stress relief. As one participant stated, “*If I am full of stress this bar and video hall will help relieve my stress*” (Esther, 21 years). However, excessive drinking, noise, and disorder were also said to generate emotional distress for both individuals and neighbors. Participants expressed concern about instability, illness, and mental disturbance linked to heavy alcohol use, as well as anxiety about children’s exposure to bar environments. As one participant noted, “*The people living around the bars get mental disturbance because the bar produces noise from people watching football and loud music and also get worried about children learning bad habits*” (Hopebella, 19 years). Together, these accounts show that alcohol-related spaces were experienced differently across the community: while some participants viewed them as places for stress relief, recreation, and social connection, others described them as sources of noise, disruption, worry, and psychological strain.

### 3.6. Community Resilience and Economic Activity

Despite the risks described, participants acknowledged that alcohol-related businesses provide employment and income within the community. Bars were seen as generating opportunities for bar owners, attendants, vendors, and surrounding enterprises. As one participant reflected, “*There are many employment opportunities in this bar, the person who owns the pool table earns, the person selling beer earns, and also the restaurants around this area will earn some money*” (Mbampe, 18 years). Participants also described resilience in terms of business owners’ persistence despite the instability associated with alcohol-related commerce. As Lisa explained, “*Bars are risky businesses. The owner of the bar makes losses sometimes… but he does not give up; he continues to work despite these challenges*” (Lisa, 18 years). Others noted that bars could attract restaurants, food vendors, and other nearby enterprises, extending their economic effects beyond the bar itself. Participants’ accounts suggested that these businesses represented pragmatic responses to limited livelihood options, allowing community members to derive income from spaces that were otherwise associated with social and health concerns. At the same time, these economic benefits were portrayed as inseparable from associated harms, including violence, disorder, and reduced productivity. Alcohol commerce was therefore framed as both livelihood and liability, reflecting structural tensions between economic survival and community well-being.

Across all six themes, participants portrayed alcohol environments as complex social and economic spaces that were embedded within daily life, safety, and well-being. Alcohol was normalized and economically embedded, yet closely intertwined with unemployment, insecurity, financial strain, and psychosocial distress. Participants’ accounts highlight the complex and sometimes contradictory role of alcohol-related environments: the same places that provided income and social connection were also perceived as contributing to risk and harm.

## 4. Discussion

In this exploratory qualitative study, alcohol emerged as a salient environmental concern within a broader Photovoice inquiry into young women’s mental health and well-being. Participants described alcohol-related spaces as everyday social, economic, and psychosocial contexts connected to their safety, well-being, and lived experiences in a low-resource urban setting.

Their accounts highlighted the ambivalent role of alcohol-related spaces in community life. Although these spaces supported recreation, social interaction, and livelihoods, participants also associated them with insecurity, financial strain, psychological distress, and other individual and community risks that may exacerbate health inequities [13]. These findings complement our prior quantitative research documenting exposure to alcohol marketing and associations with alcohol use by showing how young women interpreted the same spaces as sources of both opportunity and harm. Alcohol environments emerged as a salient pathway through which young women described everyday experiences of safety, stress, and mental well-being in an urban informal settlement, consistent with recent research documenting high levels of comorbid alcohol use and mental health concerns among young women in Kampala [47].

Through the Photovoice method, participants described alcohol as easily accessible, highly visible, and socially normalized within their neighborhoods, as noted in previous research [4,27,28,29,30]. Public alcohol consumption, unregulated bars, and informal drinking spots were associated in participants’ accounts with feelings of insecurity and exclusion, reinforcing gender disparities in alcohol-related harm and restricting women’s mobility. Although participants linked alcohol-related spaces with both temporary stress relief and emotional strain, this exploratory study cannot determine the direction of these relationships, which may be reciprocal and shaped by broader social and economic conditions.

One key theme was alcohol’s deep integration into community life. Participants described how alcohol consumption often accompanied leisure activities like watching football, playing pool, or attending social gatherings [48]. While some appreciated these as outlets for stress relief and community bonding, others viewed them as contributing to early exposure, poor decision-making, and negative role modeling, particularly for youth. The perceived effects also differed depending on one’s relationship to the space. Patrons could experience bars and entertainment venues as places of recreation, stress relief, and social connection, while nearby residents described noise, disorder, concern for children, and psychological strain. Thus, the same alcohol-related setting could support perceived well-being for some community members while undermining it for others.

Alcohol-related businesses played a prominent economic role in participants’ accounts. Bars and entertainment venues supported a wider local network of owners, attendants, pool-table operators, food vendors, restaurants, and other nearby enterprises. Participants also described business owners’ continued operation despite fights, property damage, and financial losses as a form of persistence in a setting with limited livelihood opportunities. At the same time, these venues were associated with violence, harassment, and public disorder, suggesting that their economic benefits were accompanied by substantial insecurity and harm. This tension is consistent with research among women beer promoters in Nigeria, where relatively attractive wages drew young women into precarious work that exposed them to transportation risks, sexual harassment, and unwanted advances from male customers [49]. Related research among young Nigerians found that alcohol promotions, including discounts, quantity deals, celebrity endorsements, and sports marketing, encouraged product experimentation, impulse purchases, and heavy drinking [15]. Together, these findings show how livelihood opportunities and commercial strategies can sustain alcohol economies while increasing gendered, social, and health risks.

These economic tensions extended beyond alcohol-related businesses into households and surrounding communities. Participants described how some male family members prioritized alcohol over household needs, contributing to financial strain, tension, and emotional stress. They also associated the constant noise and disorder surrounding bars with psychological strain among adults and children. These accounts are consistent with broader global concerns that alcohol environments may intensify mental health vulnerabilities, particularly among women living in already stressed settings [18,19,50,51,52,53,54,55]. At the community level, participants also linked alcohol use with crime, violence, and insecurity. They described how unemployment, economic precarity, idle time, and substance use intersected with theft, physical altercations, and harassment. Although alcohol was sometimes used as a coping mechanism [11], its widespread presence was also associated with aggression and public disorder. For young women, these conditions shaped daily routines, mobility, and perceptions of safety, illustrating how alcohol environments intersect with gendered access to public space.

Taken together, these exploratory findings identify several priorities for future research and prevention, including alcohol availability and marketing, enforcement of existing policies, community-based approaches, and livelihood opportunities for young people, consistent with recommendations from the World Health Organization and others [56,57,58,59,60,61]. Larger, purpose-designed studies are needed to determine whether and how these approaches may reduce alcohol-related harm in similar settings. Although based on a small, site-specific sample, this study demonstrates the value of participatory methods such as Photovoice for eliciting community-based insights and centering young women’s perspectives. These insights may help inform locally grounded research and intervention development. Future studies should include larger and more diverse samples across multiple sites to examine how experiences of alcohol environments vary across groups and relate to safety, livelihoods, and well-being.

### Limitations

While this exploratory study offers contextual insights into the ways alcohol environments may shape the experiences of young women and their communities through the Photovoice method, several limitations should be considered. First, the study sample consisted of only five young women from one study site in Kampala, Uganda, and the analyses focused on a subset of the broader Photovoice dataset. Consequently, the findings may not fully capture the experiences of all young women in similar contexts across Uganda or beyond. Alcohol was not an a priori focus of the broader Photovoice project but emerged as a salient environmental concern through participants’ narratives about mental health and well-being. The Makindye site was the only site where alcohol-related photographs and narratives emerged prominently. Moreover, the study protocol did not include any alcohol-specific prompts. In retrospect, it would have been helpful to explore this topic more systematically across all sites. As a result, the findings reflect the views of a small number of participants who discussed these issues over several weeks and should be interpreted as exploratory and context-specific rather than broadly generalizable. Additionally, because alcohol was an emergent focus within a small, site-specific subset of the broader Photovoice project, the study was not designed to assess thematic saturation. The six themes should therefore be understood as descriptive categories used to organize the range of issues raised in participants’ accounts rather than as evidence of prevalence or generalizability beyond this group.

Second, only two alcohol-related photographs were available for this analysis. The analysis therefore relied primarily on participants’ captions and group-discussion narratives, with the photographs serving as contextual visual material rather than as a separate visual dataset. A study designed specifically to examine alcohol environments could have incorporated more targeted prompts and additional opportunities for participants to generate and discuss relevant photographs.

Third, the women recruited into the study may differ from women not seeking any services or not engaged with UYDEL. Although UYDEL serves young women who have limited access to education, their counterparts in the community likely also experience poverty and similar life circumstances. However, we could not assess how participants differed from nonparticipants. Fourth, the findings pertain uniquely to women. Young men and other community members may experience and interpret alcohol-related environments differently. Fifth, participation was voluntary, which may have introduced selection bias. Those who chose to take part may have had a stronger interest in the mental health topic of the project or different experiences compared to those who opted not to participate. Sixth, we do not know whether participants used alcohol and whether those consuming alcohol might have different perceptions from those who may not drink alcohol. Participants’ interpretations may therefore have varied according to personal drinking experiences that were not assessed. Seventh, although bilingual facilitators were employed to reduce language barriers, discussions were primarily conducted in English with occasional translation into Luganda. As a result, some nuances in participants’ narratives may have been altered or lost in translation, potentially affecting the interpretation of their experiences. This study also relied on self-reported accounts, which may be affected by recall and social desirability biases. Some participants were hesitant to photograph public spaces due to safety risks, which may have limited their ability to fully represent their lived experiences.

Finally, as with all qualitative research, interpretation may have been influenced by the researchers’ disciplinary backgrounds and involvement in the broader Photovoice project. To reduce the influence of individual assumptions, two researchers independently reviewed the materials, compared interpretations, and resolved differences through discussion, while additional team members brought complementary perspectives to the analysis. The small sample and emergent focus also limited the breadth and depth of the analysis and did not permit an assessment of thematic saturation. Despite these limitations, this study provides exploratory and context-specific insights into a critical public health concern and underscores the potential value of participatory research for elevating marginalized voices in research, policy, and intervention development. Previous studies have similarly demonstrated the usefulness of Photovoice for examining complex public health issues in urban [62] and rural settings [63,64].

## 5. Conclusions

This exploratory study illustrates the value of participatory methods such as Photovoice for capturing young women’s perspectives on alcohol-related places in Kampala’s informal settlements. Participants described these spaces as supporting livelihoods, recreation, and social interaction while also associating them with insecurity, financial strain, and distress. Their accounts point to potential intersections among alcohol-related environments, poverty, unemployment, children’s exposure, inadequate regulation, and gendered vulnerability. These context-specific findings identify priorities for larger, purpose-designed studies, including further examination of children’s exposure to alcohol-related settings and alcohol prevention education [65,66], and may help inform future research and community dialogue in Uganda.

## Figures and Tables

**Table 1:** List of Questions asked during photograph discussions at the three study sites.

Does this photo represent where you work, live or learn?
2. What’s really happening in this photo?
3. Why did you take this photo? What would you like to share about this photo?
4. What does this photo tell us about your community?
5. What does this photo tell us about mental health or wellbeing in your community?
6. Does this photo reflect empowerment or TOPOWA?
7. How would you title/caption this photo in one sentence?

**Table 2:** Themes of Young Women’s Experiences of Alcohol Environments and Harm (*n* = 5).

Theme	Brief Description and Context	Illustrative Quote	Key Implications
Alcohol Use and Community Dynamics	Alcohol normalized in leisure and daily life	“I want to show that there are drunkards in our community.”	Normalization shapes youth expectations and gendered safety
Unemployment and Idleness	Limited opportunities linked to bar-centered activity	“Most idle people spend time in bars.”	Structural precarity reinforces alcohol-centered spaces
Marketing and Accessibility	High visibility and child exposure	“Children can easily access these bars…”	Early normalization, intergenerational risk
Violence and Crime	Alcohol settings linked to harassment and theft	“As a young girl… they will harass me.”	Gendered insecurity in public spaces
Mental Health	Alcohol as coping and disturbance	“If I am full of stress… it helps relieve stress.”	Ambivalent psychological role
Economic Activity	Bars provide income but create harm	“There are many employment opportunities in this bar.”	Economic reliance complicates regulation

## Data Availability

A Photovoice project report and findings can be shared upon reasonable request.

## Abbreviations

The following abbreviations are used in this manuscript:

LMICs	low- and middle-income countries
UYDEL	Uganda Youth Development Link

## References

[1] Patel V., Saxena S., Lund C., Thornicroft G., Baingana F., Bolton P., Chisholm D., Collins P.Y., Cooper J.L., Eaton J. (2018). The Lancet Commission on global mental health and sustainable development. Lancet.

[2] World Health Organization (2024). Global Status Report on Alcohol and Health and Treatment of Substance Use Disorders 2024.

[3] Kalungi H., Kamacooko O., Lunkuse J.F., Namutebi J., Naluwooza R., Price M.A., Mayanja Y. (2024). Prevalence and factors associated with illicit drug and high-risk alcohol use among adolescents living in urban slums of Kampala, Uganda. BMC Public Health.

[4] Swahn M.H., Culbreth R., Salazar L.F., Tumwesigye N.M., Jernigan D.H., Kasirye R., Obot I.S. (2020). The prevalence and context of alcohol use, problem drinking and alcohol-related harm among youth living in the slums of Kampala, Uganda. Int. J. Environ. Res. Public Health.

[5] Culbreth R., Nielsen K., Mobley K., Palmier J., Kavuma A., Kasirye R., Swahn M.H. (2026). Illegal Drug Use, Sleep, and Mental Health Amongst Young Women Living in the Slums of Kampala. Drug Alcohol Rev..

[6] Culbreth R.E., Nielsen K.E., Mobley K., Palmier J., Bukuluki P., Swahn M.H. (2024). Life Satisfaction Factors, Stress, and Depressive Symptoms among Young Women Living in Urban Kampala: Findings from the TOPOWA Project Pilot Studies. Int. J. Environ. Res. Public Health.

[7] Culbreth R., Masyn K.E., Swahn M.H., Self-Brown S., Kasirye R. (2021). The interrelationships of child maltreatment, alcohol use, and suicidal ideation among youth living in the slums of Kampala, Uganda. Child Abus. Negl..

[8] Lyons M., Swahn M.H., Whitaker D.J., Brown J., Kasper J., Culbreth R. (2021). Associations of Lifetime Prevalence of Homelessness with Risky Sex, Sexual Victimization, Depressive Symptoms, and Suicidality among Youth in Kampala, Uganda. Int. Soc. Work.

[9] Bondarchuk C.P., Magidson J., Mellins C., Lemon T.L., Rousseau E., Sindelo S., Medina-Marino A., Sibanda N., Butler L.M., Bekker L.G. (2025). Maladaptive Coping Strategies Mediate the Relationship Between Depression and Anxiety and Moderate-to-Heavy Alcohol Use in Young South Africans with HIV. AIDS Behav..

[10] Patterson A.Q., Culbreth R.E., Kasirye R., Kebede S., Bitarabeho J., Swahn M.H. (2022). Self-rated physical health, health-risk behaviors, and disparities: A cross-sectional study of youth in the slums of Kampala, Uganda. Glob. Public Health.

[11] Perry E.W., Culbreth R., Gilmore A., Self-Brown S., Kasirye R., Musuya T., Ndetei D., Swahn M.H. (2024). Violence Exposure, Self-Reported Mental Health Concerns and Use of Alcohol and Drugs for Coping among Youth in the Slums of Kampala, Uganda. Int. J. Ment. Health.

[12] Swahn M.H., Culbreth R., Tumwesigye N.M., Topalli V., Wright E., Kasirye R. (2018). Problem Drinking, Alcohol-Related Violence, and Homelessness among Youth Living in the Slums of Kampala, Uganda. Int. J. Environ. Res. Public Health.

[13] Kickbusch I., Allen L., Franz C. (2016). The commercial determinants of health. Lancet Glob. Health.

[14] Gilmore A.B., Fabbri A., Baum F., Bertscher A., Bondy K., Chang H.-J., Demaio S., Erzse A., Freudenberg N., Friel S. (2023). Defining and conceptualising the commercial determinants of health. Lancet.

[15] Dumbili E.W., Swahn M.H., Osborne A. (2025). Persuasion and impact: Alcohol marketing as a commercial determinant of health among young Nigerians. Alcohol Alcohol..

[16] Mialon M. (2020). An overview of the commercial determinants of health. Glob. Health.

[17] Sargent J.D., Babor T.F. (2020). The relationship between exposure to alcohol marketing and underage drinking is causal. J. Stud. Alcohol Drugs.

[18] Laslett A.-M., Cook M., Ramsoomar L., Morojele N., Waleewong O. (2024). Alcohol’s impact on the health and wellbeing of women in low- and middle-income countries: An integrative review. Int. J. Alcohol Drug Res..

[19] Wilson I.M., Willoughby B., Tanyos A., Graham K., Walker M., Laslett A.M., Ramsoomar L. (2024). A global review of the impact on women from men’s alcohol drinking: The need for responding with a gendered lens. Glob. Health Action.

[20] Swahn M.H., Haberlen M., Palmier J.B., Kasirye R. (2014). Alcohol and Drug Use and Other High-Risk Behaviors among Youth in the Slums of Kampala, Uganda: Perceptions and Contexts Obtained through Focus Groups. Int. J. Alcohol Drug Res..

[21] Morojele N.K., Dumbili E.W., Obot I.S., Parry C.D. (2021). Alcohol consumption, harms and policy developments in sub-Saharan Africa: The case for stronger national and regional responses. Drug Alcohol Rev..

[22] Muhwezi A., Murungi T., Musinguzi P., Akampumuza D., Samantha M., Ocan M., Ochola H., Rukundo G.Z., Maling S., Wakida E.K. (2026). Alcohol Use Disorder in Southwestern and Northern Uganda: Prevalence and associated factors. PLoS Glob. Public Health.

[23] Uganda Alcohol Policy Alliance (2022). Uganda Alcohol Report 2022. https://www.uapa.or.ug/sites/default/files/publications/FINAL%20Uganda%20Alcohol%20REPORT%202022.pdf.

[24] Rehm J., Neufeld M., Room R., Sornpaisarn B., Štelemėkas M., Swahn M.H., Lachenmeier D.W. (2022). The impact of alcohol taxation changes on unrecorded alcohol consumption: A review and recommendations. Int. J. Drug Policy.

[25] Uganda Revenue Authority Annual Revenue Performance Report FY 2019/2020 URA, 2020. https://www.scribd.com/document/512895150/Tax.

[26] Swahn M.H., Robow Z., Balenger A., Staton C.A., Kasirye R., Francis J.M., Komba S., Siema P. (2022). Preventing Alcohol-Related Harm in East Africa: Stakeholder Perceptions of Readiness across Five Countries. Int. J. Environ. Res. Public Health.

[27] Madden J., Swahn M., Kasirye R. (2024). Who is marketing alcohol in the slums of Kampala? A closer look at marketing types, content and brands. Int. J. Alcohol Drug Res..

[28] Swahn M.H., Natuhamya C., Culbreth R., Palmier J., Kasirye R., Dumbili E.W. (2025). Alcohol marketing as a commercial determinant of health: Daily diary insights from young women in Kampala. Health Promot. Int..

[29] Swahn M.H., Palmier J.B., May A., Dai D., Braunstein S., Kasirye R. (2022). Features of alcohol advertisements across five urban slums in Kampala, Uganda: Pilot testing a container-based approach. BMC Public Health.

[30] Swahn M.H., Culbreth R., Fodeman A., Cottrell-Daniels C., Tumwesigye N.M., Jernigan D.H., Kasirye R., Obot I. (2022). Heavy drinking and problem drinking among youth in Uganda: A structural equation model of alcohol marketing, advertisement perceptions and social norms. Drug Alcohol Rev..

[31] Swahn M.H., Culbreth R., Cottrell-Daniels C., Tumwesigye N., Jernigan D.H., Obot I. (2022). Social norms regarding alcohol use, perceptions of alcohol advertisements and intent to drink among youth in Uganda. Int. J. Health Promot. Educ..

[32] Swahn M.H., Culbreth R.E., Gilmore A.K., Parrott D.J., Daigle L.E., Kasirye R., Bukuluki P. (2022). Sexual Victimization, Self-Efficacy to Refuse Sex While Drinking, and Regretting Alcohol-Involved Sex among Underserved Youth in Kampala, Uganda. Int. J. Environ. Res. Public Health.

[33] Swahn M.H., Gressard L., Palmier J.B., Kasirye R., Lynch C., Huang Y. (2012). Serious Violence Victimization and Perpetration among Youth Living in the Slums of Kampala, Uganda. West J. Emerg. Med..

[34] Swahn M.H., Culbreth R., Masyn K.E., Salazar L.F., Wagman J., Kasirye R. (2021). The Intersection of Alcohol Use, Gender-Based Violence and HIV: Empirical Findings among Disadvantaged Service-Seeking Youth in Kampala, Uganda. AIDS Behav..

[35] Swahn M.H., Culbreth R., Staton C.A., Self-Brown S.R., Kasirye R. (2017). Alcohol-Related Physical Abuse of Children in the Slums of Kampala, Uganda. Int. J. Environ. Res. Public Health.

[36] Walls H., Cook S., Matzopoulos R., London L. (2020). Advancing alcohol research in low-income and middle-income countries: A global alcohol environment framework. BMJ Glob. Health.

[37] Dhital R., Yoeli H., Adhikari A., Luitel N.P., Nadkarni A., van Teijlingen E., Sin J. (2024). Participatory asset mapping and photovoice interviews to scope cultural and community resources to reduce alcohol harm in Chitwan, Nepal. Perspect. Public Health.

[38] Letsela L., Weiner R., Gafos M., Fritz K. (2019). Alcohol Availability, Marketing, and Sexual Health Risk Amongst Urban and Rural Youth in South Africa. AIDS Behav..

[39] Vázquez M.S., Pastor A., Molina de la Fuente I., Conde Espejo P., Sureda X. (2023). Using photovoice to generate policy recommendations to improve the alcohol urban environment: A participatory action research project. Health Place.

[40] Swahn M.H., Culbreth R.E., Palmier J., Kavuma A., Jovanovic T. (2026). Applying the Research Domain Criteria to Social Determinants of Community Mental Health in Low-Resource Settings: A Contextualized Framework with Insights from the TOPOWA Study. Community Ment. Health J..

[41] McMorrow S., Swahn M.H., Palmier J., Nabulya A., Nassaka J., Bello O.O., Fritz A. (2025). A photovoice examination of community strengths and challenges in mental health and wellbeing of young women in three urban communities in Kampala, Uganda. Glob. Health Promot..

[42] Swahn M.H., Nabulya A., Nassaka J., Palmier J. (2025). Feasibility, Challenges and Lessons Learned: Photovoice Implementation Among Young Women in the Urban Slums in Kampala, Uganda. Int. J. Qual. Methods.

[43] Swahn M.H., Nassaka J., Nabulya A., Palmier J., Vaught S. (2022). A Qualitative Assessment of Place and Mental Health: Perspectives of Young Women Ages 18–24 Living in the Urban Slums of Kampala, Uganda. Int. J. Environ. Res. Public Health.

[44] Swahn M.H., Palmier J., Nabulya A., Nassaka J. (2026). Through their eyes: A Photovoice study of environmental stressors and mental health among young women in Kampala’s urban slums. Cities Health.

[45] Wang C., Burris M.A. (1997). Photovoice: Concept, methodology, and use for participatory needs assessment. Health Educ. Behav..

[46] Catalani C., Minkler M. (2010). Photovoice: A review of the literature in health and public health. Health Educ. Behav..

[47] Swahn M., Palmier J., Natuhamya C., Mobley K., Gittner K.B., Lyons M., Bbosa G.S., Matovu G., Mubiru F., Kavuma A. (2026). Prevalence and comorbidity of mental health concerns, alcohol and drug use among young women in the urban slums of Kampala, Uganda: Findings from the TOPOWA study. BMC Public Health.

[48] Alves D., Delgado A.P., Amado M., Craveiro I., Santos Z., Goggins A., Gasparinho C., Correia A., Gonçalves L. (2022). Recreation and Alcohol Consumption in Sub-Saharan Africa: Addressing Gender and Age Differences in Urban Areas-Praia, Cabo Verde. Int. J. Environ. Res. Public Health.

[49] Dumbili E.W., Nelson E.U.E. (2023). Sexualized alcohol marketing, precarious work and gendered sexual risks: Explorations of women beer promoters in Benin City, Nigeria. Drugs Educ. Prev. Policy.

[50] Cain K.S., Meyer S.C., Cummer E., Patel K.K., Casacchia N.J., Montez K., Palakshappa D., Brown C.L. (2022). Association of Food Insecurity with Mental Health Outcomes in Parents and Children: A Systematic Review. Acad. Pediatr..

[51] Baingana F., Al’Absi M., Becker A.E., Pringle B. (2015). Global research challenges and opportunities for mental health and substance-use disorders. Nature.

[52] Lien L., Hauff E., Martinez P., Eide A.H., Swarts L., Ayazi T. (2016). Alcohol use in South Sudan in relation to social factors, mental distress and traumatic events. BMC Public Health.

[53] Logie C.H., Okumu M., Admassu Z., MacKenzie F., Tailor L., Kortenaar J.L., Perez-Brumer A., Ahmed R., Batte S., Hakiza R. (2024). Exploring ecosocial contexts of alcohol use and misuse during the COVID-19 pandemic among urban refugee youth in Kampala, Uganda: Multi-method findings. J. Migr. Health.

[54] Oyekunle V., Tomita A., Gibbs A. (2023). High levels of poor mental health among young men in urban informal settlements in South Africa: A community-based study of social determinants. Psychol. Health Med..

[55] Dance S., Adams S., Weyman A., Herbert A., Burke C., Cassidy N., Higson-Sweeney N., Dack C. (2025). Psychosocial factors associated with alcohol use in lower socioeconomic position populations: A scoping review. BMC Public Health.

[56] World Health Organization (2019). The Technical Package: SAFER.

[57] Campbell C.A., Hahn R.A., Elder R., Brewer R., Chattopadhyay S., Fielding J. (2009). The effectiveness of limiting alcohol outlet density as a means of reducing excessive alcohol consumption and alcohol-related harms. Am. J. Prev. Med..

[58] World Health Organization (2018). Global Status Report on Alcohol and Health 2018. https://www.who.int/publications/i/item/9789241565639.

[59] Anderson P., Chisholm D., Fuhr D.C. (2009). Effectiveness and cost-effectiveness of policies and programmes to reduce the harm caused by alcohol. Lancet.

[60] Gard H., Isma G.E., Petersson M., Sjöblom Andersson H., Mangrio E., Enskär K., Ingvarsdotter K. (2025). Steps Toward Justice: A model for equitable involvement of young people in mental health promotion. Front. Public Health.

[61] Ibitoye M., Kaaya S., Parker R., Likindikoki S., Ngongi L., Sommer M. (2019). The influence of alcohol outlet density and advertising on youth drinking in urban Tanzania. Health Place.

[62] Ssemugabo C., Nalinya S., Lubega G.B., Ndejjo R., Musoke D. (2021). Health Risks in Our Environment: Urban Slum Youth’ Perspectives Using Photovoice in Kampala, Uganda. Sustainability.

[63] Esau D., Ho P.T., Blair G.K., Duffy D., O’Hara N.N., Kapoor V., Ajiko M. (2017). Engaging youth in rural Uganda in articulating health priorities through Photovoice. Glob. Health Promot..

[64] Groenewald C., Essack Z., Khumalo S. (2018). Speaking through pictures: Canvassing adolescent risk behaviours in a semi-rural community in KwaZulu-Natal Province, South Africa. S. Afr. J. Child Health.

[65] Nalugya J.S., Skylstad V., Babirye J.N., Ssemata A.S., Ndeezi G., Bangirana P., Engebretsen I.M.S., Nakasujja N. (2023). “She gives it to her child who doesn’t even talk”: A qualitative exploration of alcohol and drug use among primary school-age children in Uganda. BMC Public Health.

[66] Valeckaite N., Kühl M.J., Babirye J.N., Kiirya A.K., Engebretsen I.M.S. (2025). Alcohol education in Ugandan primary schools: Teaching approaches and learners’ perspectives. BMC Public Health.

